# Histidine-based ionizable cationic surfactants: novel biodegradable agents for hydrophilic macromolecular drug delivery

**DOI:** 10.1007/s13346-023-01511-8

**Published:** 2024-01-30

**Authors:** Ilaria Polidori, Dennis To, Gergely Kali, Andreas Bernkop-Schnürch

**Affiliations:** Department of Pharmaceutical Technology, Institute of Pharmacy, Center for Chemistry and Biomedicine, University of Innsbruck, Innsbruck, 6020 Austria

**Keywords:** Histidine, Biodegradable surfactants, Ionizable cationic lipids, Hydrophobic ion-pairing, DNA delivery, Lipid-based formulations

## Abstract

The aim of this study was to design surfactants based on histidine (His) for hydrophobic ion-pairing and evaluate their safety and efficacy. Lauryl, palmitoyl and oleyl alcohol, as well as 2-hexyl-1-decanol were converted into surfactants with histidine as head-group via esterification. The synthesized His-surfactants were characterized regarding pK_a_, critical micellar concentration (CMC), biodegradability, toxicity on Caco-2 cells, and ability to provide endosomal escape. Furthermore, the suitability of these agents to be employed as counterions in hydrophobic ion pairing was evaluated. Chemical structures were confirmed by ^1^H-NMR, FT-IR, and MS. The synthesized surfactants showed pK_a_ values ranging from 4.9 to 6.0 and CMC values in the range of 0.3 to 7.0 mM. Their biodegradability was proven by enzymatic cleavage within 24 h. Below the CMC, His-surfactants did not show cytotoxic effects on Caco-2 cells (cell viability > 80%). All His-surfactants showed the ability to provide endosomal escape in a pH-dependent manner in the range of 5.2 to 6.8. Complexes formed between His-surfactants and heparin or plasmid DNA (pDNA) via hydrophobic ion pairing showed at least 100-fold higher lipophilicity than the correspondent model drugs. According to these results, His-surfactants might be a promising safe tool for delivering hydrophilic macromolecular drugs and nucleic acids.

**Graphical abstract:**

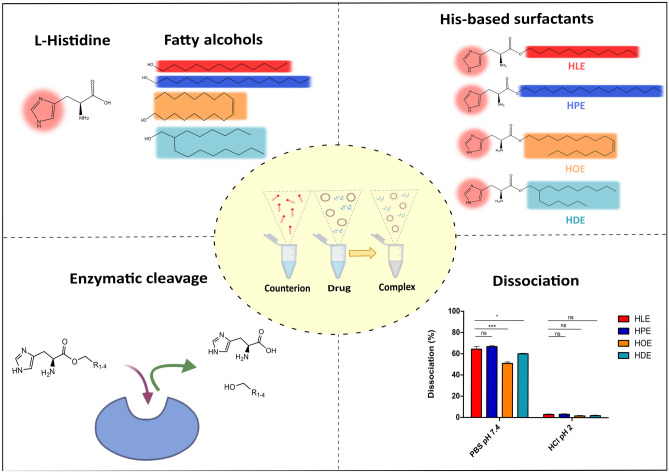

**Supplementary Information:**

The online version contains supplementary material available at 10.1007/s13346-023-01511-8.

## 1 Introduction

The term ´biopharmaceuticals´ refers to a heterogeneous class of therapeutical hydrophilic macromolecular drugs such as peptides, proteins, polysaccharides, monoclonal antibodies, and, more recently, nucleic acids [1]. The majority of these drugs are typically administered via the parenteral route. Despite overcoming stability issues related to the harsh environment in the gastrointestinal tract (GIT), the parenteral route bears disadvantages such as frequent and painful administrations leading to scarce patients’ compliance. Therefore, there is a high demand for alternative administration routes. Among all the developed drug delivery systems, lipid-based formulations (LBF) offer a potential non-invasive alternative to parenteral administration. Examples of lipid-based formulations are represented by oil-in-water nanoemulsions, self-emulsifying drug delivery systems (SEDDS), solid lipid nanoparticles (SLN), nanostructure lipid carriers (NLC), liposomes, and micelles [2]. Among those, SEDDS have already proven to provide protection against the acidic pH of the stomach [3, 4] and enzymatic degradation [3–6], improved mucus permeation properties [3, 7, 8], and cellular uptake, which in turn leads to an improved bioavailability [4–6, 8]. In order to develop a stable and effective delivery system for these hydrophilic therapeutics, it is required to incorporate such macromolecular drugs into lipophilic systems. Hydrophobic ion pairing (HIP) seems to be the most promising strategy to alter the solubility of hydrophilic drugs without chemical modification [4, 9–12]. In this approach, hydrophilic drugs bearing one or more charges are co-precipitated thanks to establishing an ionic interaction with a counterion bearing one or more opposite charges and at least one hydrophobic domain [9]. Among the different types of available counterions, surfactants are the most employed for this purpose [3, 13–17]. However, the major drawback connected to the use of these agents is that their cationic nature has been proven to be responsible for cellular, nuclear, and mitochondrial membrane destabilization [18], generation of reactive oxygen species [19] and DNA damage [20]. The negative impact of commonly employed cationic surfactants on soils, aquatic life, and humans led to the increased demand for safer and biodegradable alternatives. Ideally, the next generation of safer cationic agents is *in-vivo* degraded into non-toxic, preferably endogenous, building blocks. On the one hand, a recent solution to circumvent this problem is represented by spreading the positive charge of the cationic head by delocalizing it into a heterocyclic ring (imidazolium or pyridinium polar head) [21]. On the other hand, the design of such compounds could be inspired by naturally occurring, non-toxic compounds. Still, these new agents need to be able to form lipophilic complexes via ionic interactions with a variety of therapeutic compounds to increase their lipophilic properties, facilitating their incorporation into LBF. A couple of research groups have already investigated the safety of new cationic surfactants that bear mainly arginine and lysine as cationic head groups [22–28] and are able to be degraded into non-harmful building blocks. Nonetheless, to the best of our knowledge, no investigation has been conducted on the potential use of histidine as polar head group of counterions for hydrophobic ion pairing. The use of histidine can be advantageous due to the pK_a_ of the imidazolium ring of 6.0-6.1, which allows to obtain compounds that can bear a positive charge on the imidazolium ring only at relatively low pH values. This property can be exploited to form electrostatic interactions with a variety of therapeutics and to destabilize the endosomal membrane after cellular uptake. Therefore, this study aims to evaluate the safety of surfactants based on histidine as head group and naturally occurring fatty alcohols connected via ester bond in order to provide biodegradability. The newly produced His-surfactants were characterized regarding their physicochemical properties (CMC, pK_a_), their enzymatic biodegradability and toxicity on Caco-2 cell line, as well as their potential to allow endosomal escape. Their suitability and efficiency as hydrophobic ion pairing agents was evaluated by using heparin and plasmid DNA (pDNA) as model drugs.

## Materials

Tert-butyl-4-[(2 S)-3-(2,5-dioxopyrrolidin-1-yl)oxy-2-[(2-methylpropan-2-yl)oxycarbonylamino]-3-oxopropyl]imidazole-1-carboxylate (Boc-His(Boc)-OSu) was obtained from Bachem (Switzerland). L-histidine, lauryl alcohol, 1-hexadecanol, 2-hexyl-1-decanol, calcium chloride, sodium chloride, sodium hydroxide, sodium hydrogen phosphate, potassium dihydrogen phosphate, potassium chloride, trifluoroacetic acid, Triton-X 100, dichloromethane anhydrous, methanol, 4-dimethylaminopyridine (DMAP), potassium permanganate and trypsin from porcine pancreas (1000–2000 BAEE units/mg) were obtained from Sigma-Aldrich (Vienna, Austria). 2,2-Bis(hydroxymethyl)- 2,2′,2′′-nitrotriethanol (BIS-TRIS, ≥ 98%), heparin, and oleyl alcohol (technical grade 85%) were obtained from Fisher Scientific (Austria). The human erythrocyte concentrate was kindly provided by Tirol Kliniken (Innsbruck, Austria). All other chemicals, reagents, and solvents were obtained from commercial sources.

## Methods

### General procedure for the synthesis of his-surfactants

**Fig. 1:**
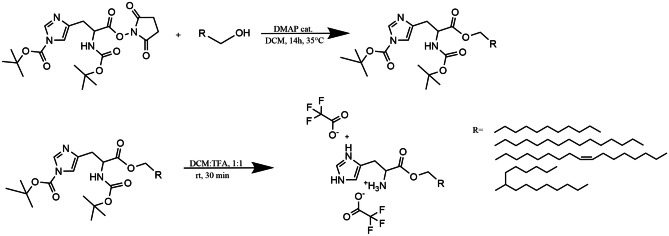
Synthesis of HLE, HPE, HOE, and HDE. *Tert*-butyloxycarbonyl (BOC), 4- dimethylaminopyridine (DMAP), dichloromethane (DCM), and trifluoroacetic acid (TFA)

His-surfactants were synthesized as represented in Fig. 1. Briefly, 1 mmol of Boc-His(Boc)-OSu, 1.2 mmol of fatty alcohol, and 0.12 mmol of 4-dimethylaminopyridine (DMAP) were dissolved in 3 mL of dichloromethane (DCM). The mixture was continuously stirred at 35 °C for 14 h. The progress of the reaction was monitored by thin layer chromatography (TLC), using a mobile phase composed of chloroform/ethyl acetate (1:1, V/V) on a 0.5 mm thick precoated silica gel aluminum sheet as stationary phase. The plate was sprayed with an aqueous solution composed of 0.5 M dipotassium carbonate and 1 M NaOH (50:14, V/V) containing 0.06 M potassium permanganate and subsequently heated to obtain visible yellow spots. After the completion of the reaction, the mixture was dried with a rotary evaporator (Heidolph VAP Value G3B, Heidolph Instruments, Germany). Subsequently, the removal of BOC protecting groups to yield the final products was achieved by dissolving the solids in 5 mL of dichloromethane/trifluoroacetic acid (TFA) (9:1, V/V) under continuous stirring. The reaction was kept at room temperature for 30 min and the progress of the reaction was monitored by TLC using precoated silica gel RP-18 W aluminum sheets using a mobile phase composed of demineralized water/acetonitrile (6:4, V/V) and 0.1% TFA. The TLC plate was sprayed with 0.5% (m/V) ninhydrin solution as detection reagent. The mixture was dried under vacuum to remove excess of TFA, and a reversed-phase column chromatography was performed in order to remove residual educts and side products. For this, silica gel C18 (pore size 60 Å, particle size 35–70 μm) was used as stationary phase, while water/acetonitrile (6:4, V/V) and 0.1% TFA was used as mobile phase. Eluted fractions in 2 mL volume were collected and analysed by TLC, as mentioned before. Afterward, the purified product was obtained from selected fractions by freeze-drying. Final products were stored at -20 °C until further use. FT-IR, ^1^H-NMR, and MS spectra of all compounds are reported in Supplementary materials (S1-S6).

#### Histidine-lauryl ester (HLE)

Boc-His(Boc)-OSu (452.46 mg, 1 mmol), lauryl alcohol (223.6 mg, 1.2 mmol) and DMAP (14.7 mg, 0.12 mmol) were dissolved in 3 mL of DCM and kept under continuous stirring for 14 h at 35 °C. Subsequently, removal of Boc groups and purification yielded a white solid (225 mg, yield 40.0%). IR *ν*_max_ 2918, 2851 (sp^3^ -CH), 1687 (ester C = O), 1139 cm^−1^ (ester C-O).^1^H-NMR (DMSO- *d*_*6*_, 400 MHz) δ = 8.87, 7.42 (2 H, 2s, histidine -C*H*=), 4.35 (1H, m, -C*H*(NH_2_)-), 4.11 (2 H, m, histidine -C*H*_*2*_-), 3.19 (2 H, m, -C(O)-C*H*_*2*_-), 1.51 (2 H, s, -C(O)-CH_2_-C*H*_*2*_-), 1.23 (18 H, s, -C*H*_*2*_-) 0.85 ppm (3H, m, -C*H*_*3*_). *m/z* = 324.1 (M^+^, 324.1%), 325.2 (10).

#### Histidine-palmitoyl ester (HPE)

Boc-His(Boc)-OSu (452.46 mg, 1 mmol), palmitoyl alcohol (290.9 mg, 1.2 mmol) and DMAP (14.7 mg, 0.12 mmol) were dissolved in 3 mL of DCM and kept under continuous stirring for 14 h at 35 °C. Subsequently, removal of Boc groups and purification yielded a white solid (336 mg, yield 60.4%). IR *ν*_max_ 2919, 2851 (sp^3^ -CH), 1689 (ester C = O), 1140 cm^−1^ (ester C-O).^1^H-NMR (DMSO- *d*_*6*_, 400 MHz) δ = 8.87, 7.42 (2 H, s, histidine -C*H*=), 4.35 (1H, m, -C*H*(NH_2_)-), 4.11 (2 H, m, histidine -C*H*_*2*_-), 3.19 (2 H, m, -C(O)-C*H*_*2*_-), 1.51 (2 H, s, -C(O)-CH_2_-C*H*_*2*_-), 1.23 (24 H, s, -C*H*_*2*_-) 0.85 ppm (3H, m, -C*H*_*3*_). *m/z* = 340.4 (M^+^, 100%), 379.6 (75), 381.1 (20).

#### Histidine-oleyl ester (HOE)

Boc-His(Boc)-OSu (452.46 mg, 1 mmol), oleyl alcohol (322.2 mg, 1.2 mmol) and DMAP (14.7 mg, 0.12 mmol) were dissolved in 3 mL of DCM and kept under continuous stirring for 14 h at 35 °C. Subsequently, removal of Boc groups and purification yielded a white solid (300 mg, yield 50.4%). IR *ν*_max_ 2923, 2853 (sp^3^ -CH), 1665 (ester C = O), 1133 cm^−1^ (ester C-O).^1^H-NMR (DMSO- *d*_*6*_, 400 MHz) δ = 8.85, 7.41 (2 H, s, -C*H*=), 5.33 (2 H, s, -C*H*=), 4.35 (1H, m, -C*H*(NH_2_)-), 4.11 (2 H, m, histidine -C*H*_*2*_-), 3.20 (2 H, m, -C(O)-C*H*_*2*_-), 1.99 (2 H, s, -C*H*_*2*_-CH=), 1.52 (2 H, s, -C(O)-CH_2_-C*H*_*2*_-), 1.23 (22 H, s, -C*H*_*2*_-) 0.85 ppm (3H, m, -C*H*_*3*_). *m/z* = 405.8 (M^+^, 100%), 403.5 (25), 407.6 (10).

#### Histidine-2-hexyl-1-decanol ester (HDE)

Boc-His(Boc)-OSu (452.46 mg, 1 mmol), 2-hexyl-1-decanol (290.9 mg, 1.2 mmol) and DMAP (14.7 mg, 0.12 mmol) were dissolved in 3 mL of DCM and kept under continuous stirring for 14 h at 35 °C. Subsequently, removal of Boc groups and purification yielded a white solid (270 mg, yield 45.4%). IR *ν*_max_ 2928, 2858 (sp^3^ -CH), 1665 (ester C = O), 1131 cm^−1^ (ester C-O).^1^H-NMR (DMSO- *d*_*6*_, 400 MHz) δ = 8.85, 7.41 (2 H, s, -C*H*=), 4.39 (2 H, m, -C*H*<-), 4.03 (2 H, m, histidine -C*H*_*2*_-), 3.19 (2 H, m, -C(O)-C*H*_*2*_-), 1.54 (2 H, s, -C(O)-C*H*_*2*_-CH<), 1.23 (24 H, s, -C*H*_*2*_-) 0.85 ppm (6 H, m, -C*H*_*3*_). *m/z* = 380.1 (M^+^, 100%), 380.8 (35), 379.5 (20).

### Chemical characterization of his-surfactants

#### FT-IR

FT-IR spectra were recorded on a Bruker ALPHA FT-IR equipped with a Platinum ATR sampling module and analysed with OPUS Spectroscopy Software, version 7. FT-IR spectra were recorded at a resolution of 4 cm^−1^ in the wavenumber range from 4000 to 400 cm^−1^.

#### ^1^H-NMR

^1^H-NMR measurements were performed on a “Mars” 400 MHz Avance 4 Neo spectrometer from Bruker Corporation (Billerica, MA, USA, 400 MHz) in dimethyl sulfoxide-*d*_*6*_ (DMSO-*d*_*6*_) solution. Chemical shifts were reported in parts per million, and deuterated solvent, DMSO-*d*_*6*_, served as the internal standard (δ 2.5 ppm).

#### MS

The molecular mass of His-surfactants was determined using MS according to a method described [28] with some modifications. Mass spectrometry detection was carried out using a Chromaster 5610 MS detector (Hitachi) controlled by a computer running the MSD system manager software (version 2.1). Analysis conditions were set as follows: ionization potential (V): 2500; counter gas flow (L/min): 0.6; AIF temperature 120 °C; ion source temperature 70 °C; API temperature 120 °C; AIF introducing energy (eV): 5.0; AIF excreting voltage (V): 5.0; AP1 voltage (V): 80.0 and AP2 voltage (V): 30.0. Sample solutions were prepared by dissolving His-surfactants in methanol at a concentration of 1 mg/mL and 2 µL were injected. Mass spectral studies were performed in positive electrospray ionization (ESI) mode in the mass range of m/z 250–400 for histidine-lauryl ester (HLE), and 300–800 for histidine-palmitoyl ester (HPE), histidine-oleyl ester (HOE) and histidine-(2-hexyl-1-decyl) ester (HDE) to determine molecular weights.

#### Determination of CMC and pK_a_

The critical micellar concentration was determined using a previously described method [28, 29]. Briefly, the surface tension of unbuffered aqueous solutions and solutions at pH 2 of HLE, HPE, HOE, and HDE at different concentrations ranging from 0.1 to 10 mM was recorded using the drop shape analysis instrument Kruess DSA25E (Kruess, D-Hamburg, Germany). Using the pendant drop technique at ambient conditions, the surface tension of all aqueous solutions was measured in series of 3 drops each. The drop was suspended from a needle (outside diameter: 1.83 mm), and the drop volume was increased by a dosimeter. The pK_a_ values of HLE, HPE, HOE, and HDE were determined by titration of 10 mL of 1 mM surfactant solution with 0.25 mM NaOH at 25 °C using a pH electrode (WTW SenTix Mic). The inflection point was determined by interpolating the points and smoothing the curve and the corresponding pK_a_ values were calculated as the semi-equivalent point of the neutralization curve [29].

### HPLC quantification of his-surfactants

The system consisted of a Hitachi Chromaster (Tokyo, Japan) equipped with a 5160 pump, 5260 autosampler, 5310 column oven, and 5430 photodiode array UV detector. In brief, the stationary phase was a Primesep 100 column (150 × 4.6 mm, 5 μm), the column oven was set to 30 °C. For isocratic elution ACN/H_2_O (90:10, V/V) containing H_2_SO_4_ 0.2% V/V with a flow rate of 1.5 mL/min was used as mobile phase. The detection wavelength was set to 220 nm.

### Biodegradation studies

The biodegradability of the newly synthesized His-surfactants was evaluated by applying a slightly modified method described by Strader et al. [30]. Briefly, HLE, HPE, and HOE were dissolved in 100 mM HEPES buffer pH 6.8 at a concentration of 1 mg/ml. In an Eppendorf tube, 400 µL of the surfactant stock solution was mixed with 500 µL of 100 mM HEPES buffer pH 6.8 and 100 µL of a trypsin stock solution at a concentration of 1 mg/ml prepared in 1 mM HCl. Samples were incubated for 24 h and continuously agitated at 300 rpm. At predetermined time points (10 min, 30 min, 1 h, 2 h, 4 h, and 24 h), an aliquot of 50 µL was withdrawn from every tube and 1 µL of TFA was immediately added to stop the enzymatic degradation. The amount of remaining surfactant was evaluated by HPLC quantification as described above.

### Toxicity studies

Caco-2 cells were seeded at a density of 2.5 × 10^4^ cells per well in 24 well plates. During the growth period, cells were stored in minimum essential medium (MEM) at 37 °C, 95% relative humidity, and 5% CO_2_. MEM was supplied with 10% (V/V) heat-inactivated fetal bovine serum (FBS) and penicillin/streptomycin solution (100 units/0.1 mg/L). Cells were cultured until confluency of 80–90% was obtained, and the medium was replaced every two days until the day of the experiment. Test solutions of HLE, HPE, HOE, and HDE were prepared in concentrations of double the CMC, at the CMC and half the CMC, respectively, in sterile 25 mM HEPES buffered saline (HBS) composed of 20 mM HEPES, 1 g/L glucose anhydrous, 136.7 mM NaCl, 5 mM KCl and 1 mM CaCl_2_, pH 7.4. Before applying the samples, cells were washed twice with pre-heated HBS at 37 °C. Test solutions, negative control (HBS), and positive control (0.1% V/V Triton X-100) were added in triplicate to the cell culture plate in the volume of 0.5 mL/well and incubated at 37 °C in a 5% CO_2_ environment for 4 h. After incubation, cells were washed again three times. Subsequently, HBS was replaced with a 0.1% (m/V) resazurin solution in HBS and incubated for 3 h. Cell viability was determined photometrically at an excitation wavelength of 540 nm and an emission wavelength of 590 nm. Cell viability was calculated according to the following equation:

$$Cell\, viability\, \left(\%\right):\frac{F \left(sample\right)-F\left(negative\, control\right)}{F\left(positive\, control \right)-F \left(negative\, control\right)}\times 100$$

### Endosomal Escape studies

The capacity of His-surfactants to induce pH-dependent disruption of lipid bilayer membranes was assessed via a red blood cell hemolysis assay following a slightly modified method described in the literature [31]. Briefly, surfactants were incubated for 1 h at 37 °C in the presence of human erythrocytes at concentrations ranging from 5 to 50 µg/mL in 100 mM sodium phosphate buffer (supplemented with 150 mM NaCl) in the pH range of the endosomal processing pathway (7.4, 7.0, 6.6, 6.2, and 5.8). The extent of cell lysis was determined spectrophotometrically by measuring the amount of hemoglobin released at 415 nm and normalized to a 100% lysis control (0.1% V/V Triton X-100).

### Preparation of hydrophobic ion pairs

Heparin and pDNA were used as model drugs for HIP. The plasmid DNA pcDNA3-EGFP was amplified in *Escherichia coli* cells and purified using a plasmid-extraction kit from Qiagen. The purified plasmid was dissolved in nuclease-free water and stored at − 20 °C until use. The concentration was determined using the NanoQuant Plate (Tecan Spark®, Tecan Trading AG, Zurich, Switzerland), and a gel electrophoresis was performed as control. Surfactant solutions in different concentrations were prepared in aqueous solution of HCl 0.01 M. The individual solution was added dropwise to the same volume of heparin solution (1 mg/mL) at pH 2.0 or pDNA (1 mg/mL) at pH 8.0 resulting in molar ratios ranging from 1:2 to 1:50 (heparin:surfactant) or from 1:6159 to 1:18477 (pDNA:surfactant). Solutions of heparin/pDNA and pure surfactant solutions in the same concentrations served as controls. Afterward, mixtures were incubated at room temperature for 15 min while gently shaking on a thermomixer at 300 rpm. Subsequently, HIPs and supernatants were separated by centrifugation at 12,500 rpm for 15 min at 4 °C. The obtained HIP complexes were washed twice with water, dried under vacuum, and stored at -20 °C until further use. Precipitation efficiency was calculated using the following equation:

$$Precipitation\; efficiency\; \left(\%\right)=100-\left(\frac{drug\; concentration\; after\; HIP}{drug\; concentration\; before\; HIP}\right)\times 100$$

#### Precipitation efficiency of heparin

The remaining heparin in the supernatants was quantified as described by Liu et al. [32]. Briefly, 0.001 M TBO solution was prepared in 0.03 M sodium chloride in 0.01 M HCl. Supernatant and TBO solutions were mixed in equal volume ratio and incubated at 37 °C and 300 rpm for 4 h. Heparin and TBO complex precipitated in the mixture and was separated by centrifugation at 6000 rpm for 10 min. The precipitate was carefully washed three times with 0.03 M sodium chloride in 0.01 M HCl and re-dissolved in the mixture of 80% ehanol and 0.1 M NaOH (4/1, V/V). The absorbance of the solution was measured at 530 nm. Heparin concentration was calculated from the calibration curve constructed using known concentration range of heparin (0.25–0.03 mg/mL) by TBO assay in the same manner.

#### Precipitation efficiency of pDNA

The amount of the precipitated pDNA was calculated through the remaining pDNA in the supernatant spectrophotometrically determined using the NanoQuant Plate (Tecan Spark®, Tecan Trading AG, Zurich, Switzerland) at a wavelength of 260 nm.

### Characterization of hydrophobic ion pairs

#### Partition coefficient

HIPs were further characterized regarding their partition coefficient in water and octanol (logP _n−octanol/water_). His-surfactant solution (200 µL, 1:20 or 1:18477 molar ratio) was slowly added dropwise to heparin or pDNA aqueous solution (200 µL, 1 mg/mL) at room temperature. Demineralized water added to heparin or pDNA aqueous solution served as control. After five minutes, octanol (400 µL) was added to the mixture and samples were placed on a thermomixer at 300 rpm and 25 °C for 24 h. The two phases were separated by centrifugation for 15 min at 12,500 rpm. Aliquots of 100 µL were withdrawn from each octanol phase, diluted with 100 µL of 0.1 M NaOH, and vortexed for 20 min to break the ionic interaction. Heparin was quantified via TBO assay as described above after the addition of 100 µL of 0.1 M HCl to neutralize the excess of NaOH. The amount of pDNA in water and octanol phase was determined as described above. The logP was calculated according to the following equation:

$$logP=\text{l}\text{o}\text{g}\left(\frac{Concentration\;of\;drug\;in\;octanol}{Concentration\;of\;drug\;in\;water}\right)$$

#### Dissociation

The stability of HIPs was assessed by evaluating the dissociation behavior under physiological conditions. HIPs were dispersed in PBS pH 7.4 and 0.01 M HCl pH 2.0 in concentrations of 1 mg/mL [28] and incubated for 4 h at 37 °C while shaking on a thermomixer at 300 rpm. Subsequently, mixtures were centrifuged for 15 min at 12,500 rpm, and aliquots of 100 µL were withdrawn from the supernatant and free heparin was quantified via TBO assay, pDNA was spectrophotometrically quantified as described above.

### Statistical data analysis

Statistical data analysis was performed on GraphPad Prism (version 5.1) using the student t-test and the analysis of variance (ANOVA) followed by Bonferroni correction with p ≤ 0.05 as the minimal level of significance. All values are expressed as means ± SD.

## Results and discussion

### Synthesis of His-surfactants

Four His-surfactants were successfully prepared via esterification reaction between the starting materials Boc-His(Boc)-OSu and lauryl alcohol (HLE), palmitoyl alcohol (HPE), oleyl alcohol (HOE), and 2-hexyl-1-decanol (HDE) as illustrated in Fig. 1. Boc-protected histidine was employed in order to avoid polymerization of the amino-acid and unwanted reactions on the imidazolium ring. The FT-IR, ^1^H-NMR and MS spectra of the synthesized surfactants are presented in the Supplementary Information (Figs. S1-S6). C = O vibrations were observed between 1650 and 1710 cm^−1^ for all His-surfactants. The OH stretching at 3300 cm^−1^, typical of alcohols, is not evidenced in the final products. Between 1100 and 1300 cm^−1^ the C-O stretching bands are evident, confirming the presence of an ester bond with the carboxylic function of histidine (Figs. S1-S4). The imidazole functionality of histidine can be detected around 7.4 and 8.8 ppm. The methine and methylene protons in the histidine head group present peaks at 4.39 and 3.19 ppm, respectively. The methylene groups next to the ester show a chemical shift at 4.11 ppm in the cases of HLE, HPE and HOE, while this peak is a bit downfield to 4.03 ppm in the case of HDE due to the neighboring methine. The hydrophobic fatty acid tail has peaks around 0.85 and 1.23 ppm, belonging to the methyl and methylene protons, respectively. In the case of HOE, the peak at 5.33 ppm belong to the protons of the double bond, while at 1.99 ppm to the neighboring methylenes in the fatty acid chain (Fig. S5). The MS spectra (Fig. S6) showed the mass of the final products, thereby further confirming the identity and purity of the obtained compounds.

### Characterization of His-surfactants

#### Determination of CMC and pK_a_

**Fig. 2:**
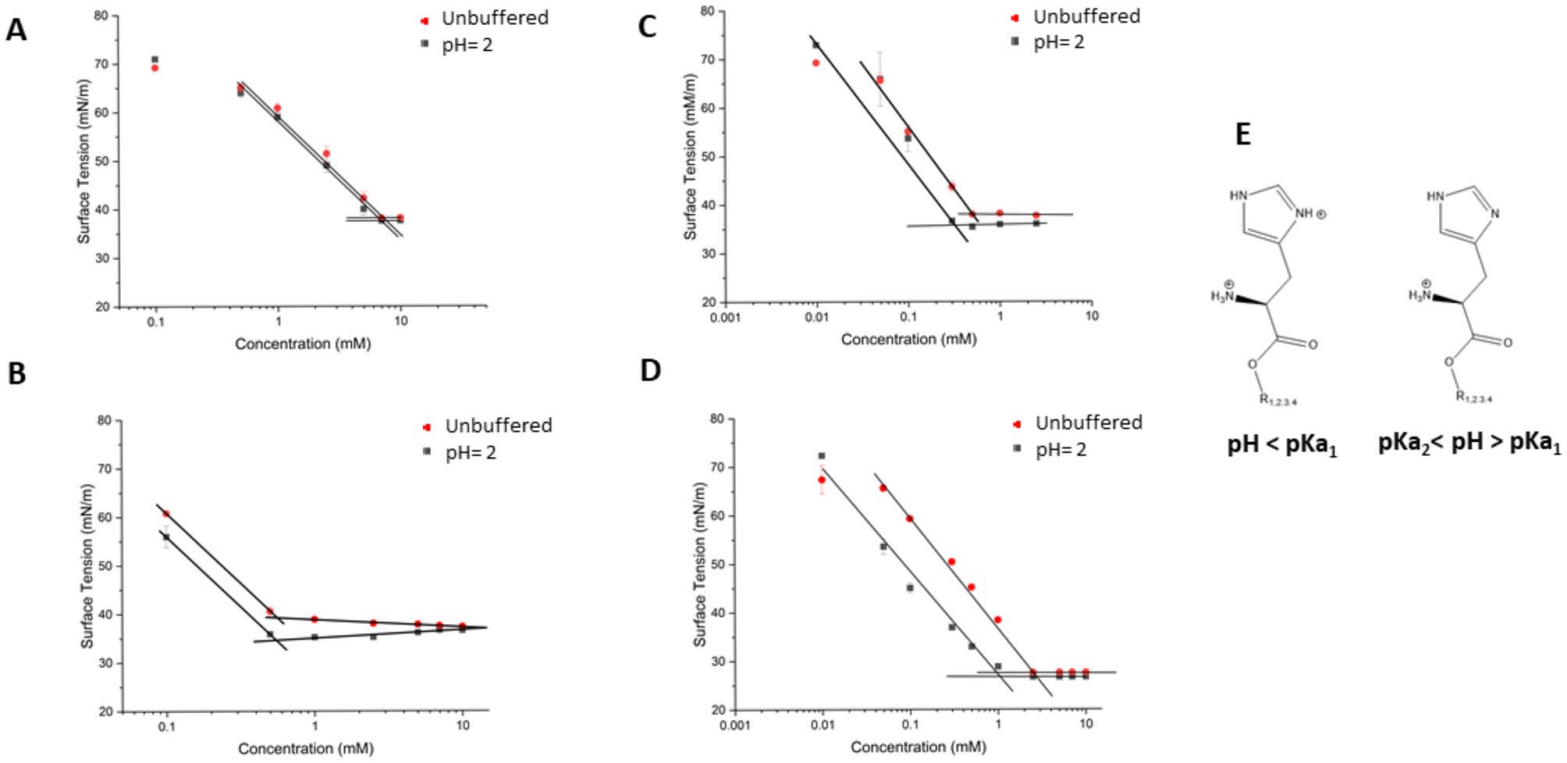
Plot of surface tension (mN/m) versus surfactant concentration (mM) for **A** HLE, **B** HPE, **C** HOE, and **D** HDE. **E** Illustration of His-surfactants charges at different pH values. Indicated values are means (n ≥ 3) ± SD

The CMC of His-surfactants was determined as a function of concentration, as shown in Fig. 2. It is well known that the micellar behaviour and, consequently, the CMC value in the case of ionizable surfactants is highly influenced by the number of displayed charges per molecule [29]. Therefore, the determination was executed at two different conditions: unbuffered solutions, where the surfactants may only bear one charge on the primary amine, and at pH 2.0, where both the primary amine and the imidazolium ring are protonated. As reported in Fig. 2, the two plots show a similar trend for every surfactant, implying that the molecules display the same number of positive charges in both conditions. The CMC was determined as the point where the increase in the surfactant concentration did not further reduce the surface tension. All His-surfactants investigated in this work can reduce the surface tension water/air at small concentrations in the following order: HLE < HPE < HDE < HOE.

In order to determine the pK_a_ of the imidazolium function of the surfactants, 10 mL of 1.0 mM surfactant solution was titrated with 2.5 mM NaOH (Fig. S7-S10). The measured pK_a_ values fall in the range of 4.9-6.0, which differ around one unit from the described pK_a_ values for the histidine imidazolium function in literature (Table 1). A possible explanation for this behaviour can be provided by considering the inductive effect exerted by the lipophilic tails. Another explanation is that the formation of micelles is known to cause pK_a_ shifts [29]. In the latter case, differences in the polarity of the bulk solvent and the interfacial region cause a change in the pKa of the solubilized species, as described by El Seoud [33]. In fact, the pK_a_ values, with the only exception of HLE, have been determined by titration of a surfactant solution at a concentration above the CMC.

Surfactants are amphiphilic molecules and, due to their nature, have a spontaneous tendency to accumulate at interfaces resulting in an inaccurate determination of the partition coefficient between water and octanol. Moreover, surfactants tend to form supramolecular aggregates at a concentration above the CMC, and this can lead to considerable overestimation of the surfactant concentration in the aqueous phase. As Short et al. reported, if the concentration is sufficiently high (i.e., > 10^−2^ M) but still below the CMC of the surfactants, the logP can be accurately determined via the classic shake flask method [34]. In this case, the CMCs determined for the His-surfactants fall in the range of 0.25 to 6 mM (Table 1), consequently, this consideration is not applicable. For the above-mentioned reasons, in this work, only logP values utilizing XLOGP3 software are reported (Table 1). Since surfactants are often used as counterions for hydrophobic ion pairing with hydrophilic drugs in order to increase their lipophilicity for incorporation in lipophilic nanocarrier systems, a comparatively high logP is advantageous. The obtained values are in the range 4.63–7.27, similarly to already reported values in literature for other cationic counterions used in hydrophobic ion pairing [35–40].

**Table 1:** Physicochemical properties of HLE, HPE, HOE, and HDE.

Compound	Formula	Molecular Weight (g/mol)	cLogP	Apparent pK_a_ (imidazolium)	CMC (mM) unbuffered	CMC (mM) pH = 2
HLE	C_18_H_33_N_3_O_2_	323.26^a^	4.63^b^	6.0	6.00	6.54
HPE	C_22_H_41_ N_3_O_2_	379.32^a^	6.80^b^	4.9	0.54	0.57
HOE	C_24_H_43_ N_3_O_2_	405.34^a^	7.27^b^	5.1	0.25	0.36
HDE	C_22_H_41_ N_3_O_2_	379.32^a^	6.83^b^	5.2	0.75	1.00

^a^Calculated with ChemDraw

^b^Calculated with XLOGP3

#### Enzymatic biodegradation of his-surfactants

**Fig. 3:**
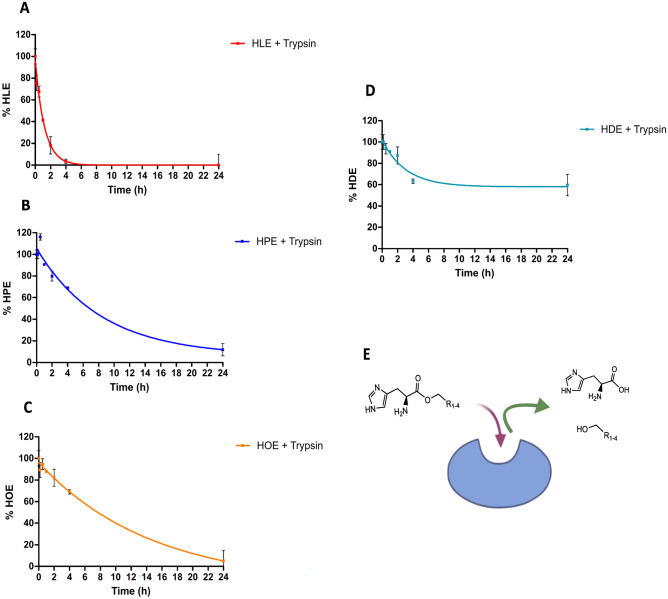
Enzymatic degradation of **A** HLE, **B** HPE, **C** HOE, and **D** HDE with trypsin (1000–2000 BAEE units mg^−1^) in 100 mM HEPES buffer at pH 6.8 Indicated values are means (n ≥ 3) ± SD

Trypsin was chosen as the model endogenous enzyme to evaluate the biodegradation of His-surfactants. Overall, the degradation of His-surfactants was completed within 24 h, with the only exception of HDE (Fig. 3). In the case of HDE, approximately 40% could be degraded within the first 4 h and no degradation was observed after this time point. Since 2-hexyl-1-decanol is most hydrophobic showing an aqueous solubility of 0.001 g/L, as specified by the manufacturer, it will likely co-precipitate with the conjugate during the cleavage process. Consequently, not the entire substrate could be degraded by trypsin. HLE ester bond was rapidly cleaved, showing a half-life of 1 h. The His-surfactants showed the following order of stability towards enzymatic degradation: HLE < HPE < HOE < HDE, supporting the role also of the lipophilicity and bulkiness of the alkyl chain in preventing enzymatic cleavage. Therefore, the outcome of the study encourages the use of these compounds for pharmaceutical applications because of the ability that endogenous enzymes, such as trypsin, have to cleave the ester bond and break down these compounds into naturally occurring, non-toxic building blocks.

#### Cytotoxicity

**Fig. 4:**
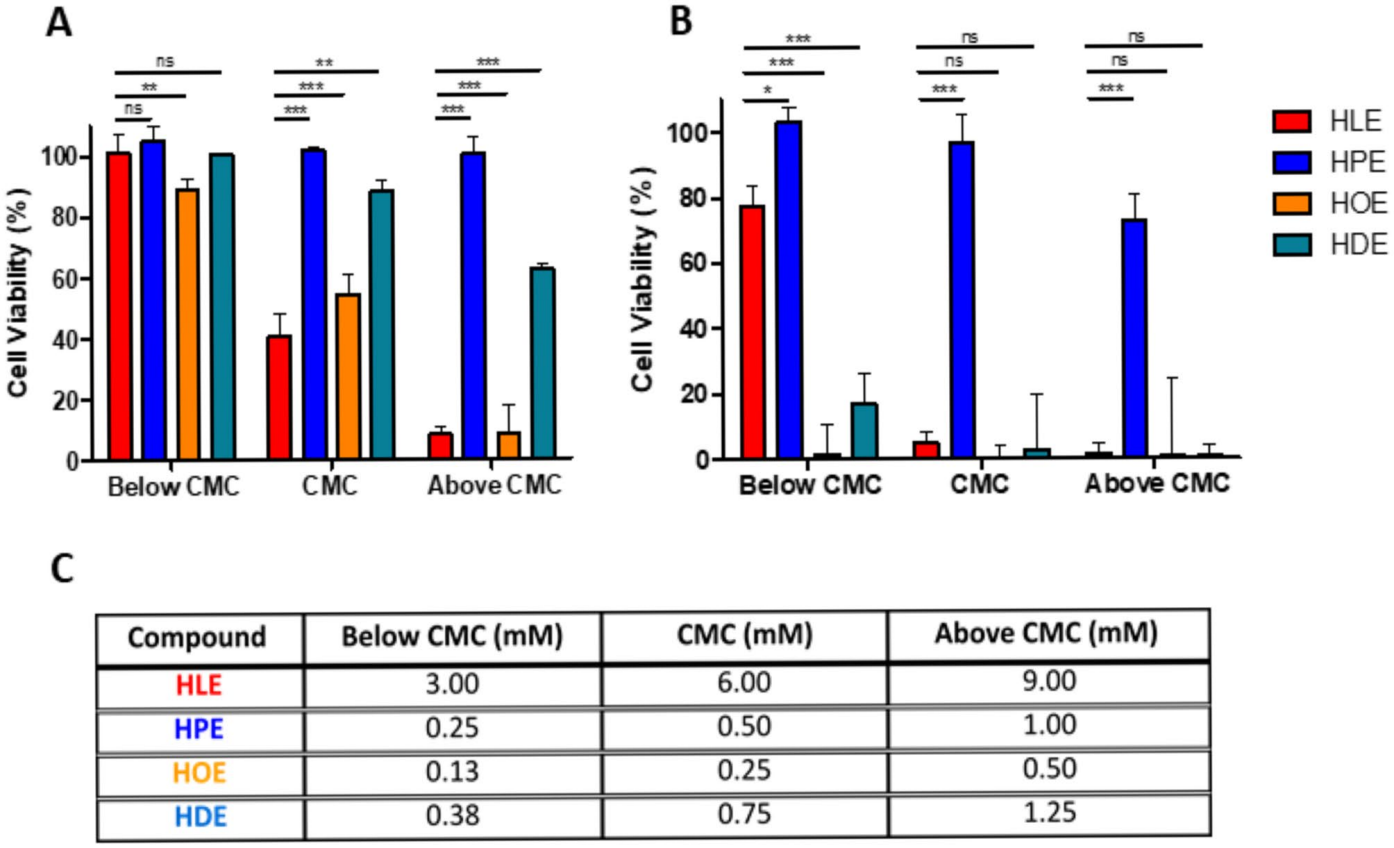
Cytotoxicity studies on Caco-2 cells of HLE (red bars), HPE (blue bars), HOE (orange bars), and HDE (turquoise bars) after **A** 4 h and **B** 24 h incubation at 37 °C using resazurin assay. **C** Concentration tested for each surfactant. Data are shown as mean ± SD (n = 3)

In order to further confirm a reduced cytotoxic potential of the newly produced surfactants, resazurin assays on Caco-2 cells were performed. Within this assay, metabolically active cells reduce the nonfluorescent dye resazurin to the highly fluorescent resorufin enabling the assessment of cell viability. The results are shown in Fig. 4. Below the CMC, every tested molecule showed cell viabilities > 80% and was considered as non-cytotoxic. At the CMC, HLE and HOE proved to be cytotoxic to some extent (cell viabilities of 40.6% and 53.9%, respectively); at a concentration twice as high as the CMC, also HDE showed a cytotoxic effect with cell viability of 62.4%. In general, two main mechanisms can explain surfactants cytotoxicity: osmotic lysis and solubilization mechanism. The first is predominant at concentrations below the CMC, while the second prevails at concentrations above the CMC [41]. Aside from the concentration, two structure-related factors strongly influence the surfactants-mediated cytotoxicity: the cationic charge density and hydrophobicity. Given the fact that these agents bear a biocompatible group with reduced cationic charge density as polar heads, the alkyl chain hydrophobicity seems to play a key role in determining their cytotoxicity. According to the proposed mechanism, HLE should be in theory less cytotoxic than HPE, HOE, and HDE, which is in contradiction to the results obtained. However, this might be explained by the around 30-fold higher concentration of HLE that was necessary because of the tremendously higher CMC value compared to the other surfactants having been tested.

#### Ability to provide endosomal escape

**Fig. 5:**
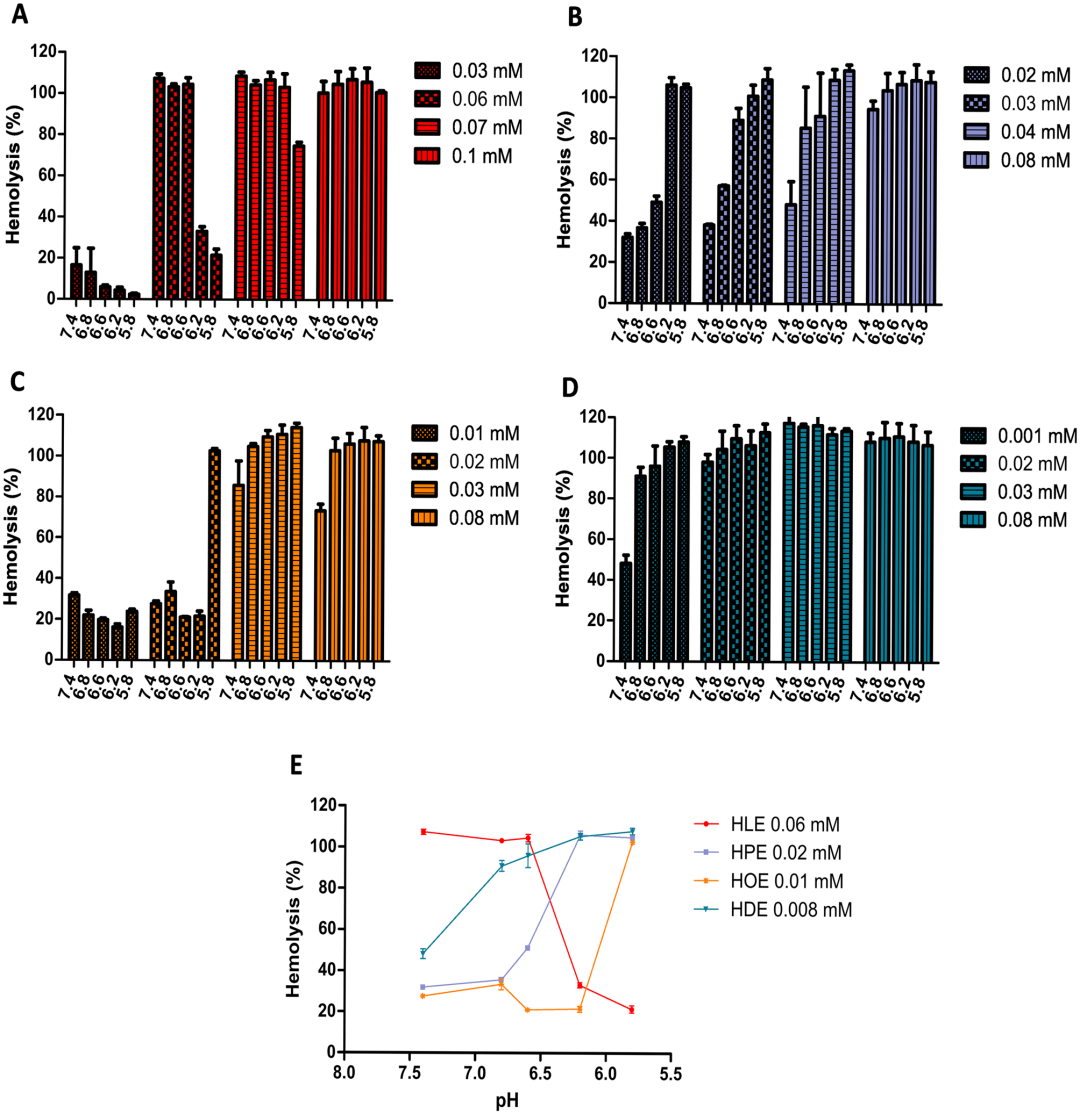
Hemolytic activity of His-surfactants. **A** Dose-dependent hemolysis of HLE. **B** Dose-dependent hemolysis of HPE. **C** Dose-dependent hemolysis of HOE. **D** Dose-dependent hemolysis of HDE. **E** Examples of pH dependent hemolysis of His-surfactants. Data are shown as mean ± SD (n = 3)

In order to confirm the applicability of His-surfactants investigated within this study as efficient drug delivery tools, the capacity of His-surfactants to provide endosomal escape was studied. Since the erythrocyte membrane is a well-established model to study surfactant-endosomal membrane interactions [42], hemolytic activity of His-surfactants was investigated over concentrations ranging from 0.001 to 0.1 mM, as a function of pH (Fig. 5). All surfactants showed a concentration-dependent increase of hemolytic activity, and this behavior is in agreement with the wide literature reporting that surfactants at high concentrations are generally hemolytic [43, 44].

A pH-dependent membrane disruption has to be achieved to allow intracellular trafficking of therapeutic compounds. Endosomal compartments have a pH of 5.5–6.2, depending on the endosomal stage [45, 46]. Therefore, a preferential destabilization or solubilization of the endosomal membrane at pH 5.5–6.8 is desirable to allow surfactant–drug complexes to escape to the cytoplasm. On the other hand, low hemolytic activity is expected at the pH range of 7.4–6.8. HPE and HOE met this requirement, showing pH-dependent increase in hemolytic activity in the desired range (Fig. 5E). This observation opens the possibility of studying these His-surfactants for these potential uses.

HDE also proved to be efficient in providing endosomal escape due to the high hemolysis at pH 5.5–6.2 but also shows pronounced hemolysis at physiological pH. Surprisingly, HLE showed an opposite behavior as expected. In the case of HPE, HOE, and HDE hemolysis increased as pH reaches values closer to pK_a1_ (Fig. 5E). In the case of HLE, the positive charge bearded by the imidazolium ring seems to exert a stabilizing effect on the erythrocyte membrane, similarly to previous observations [47–49].

### Hydrophobic ion pairing

**Fig. 6:**
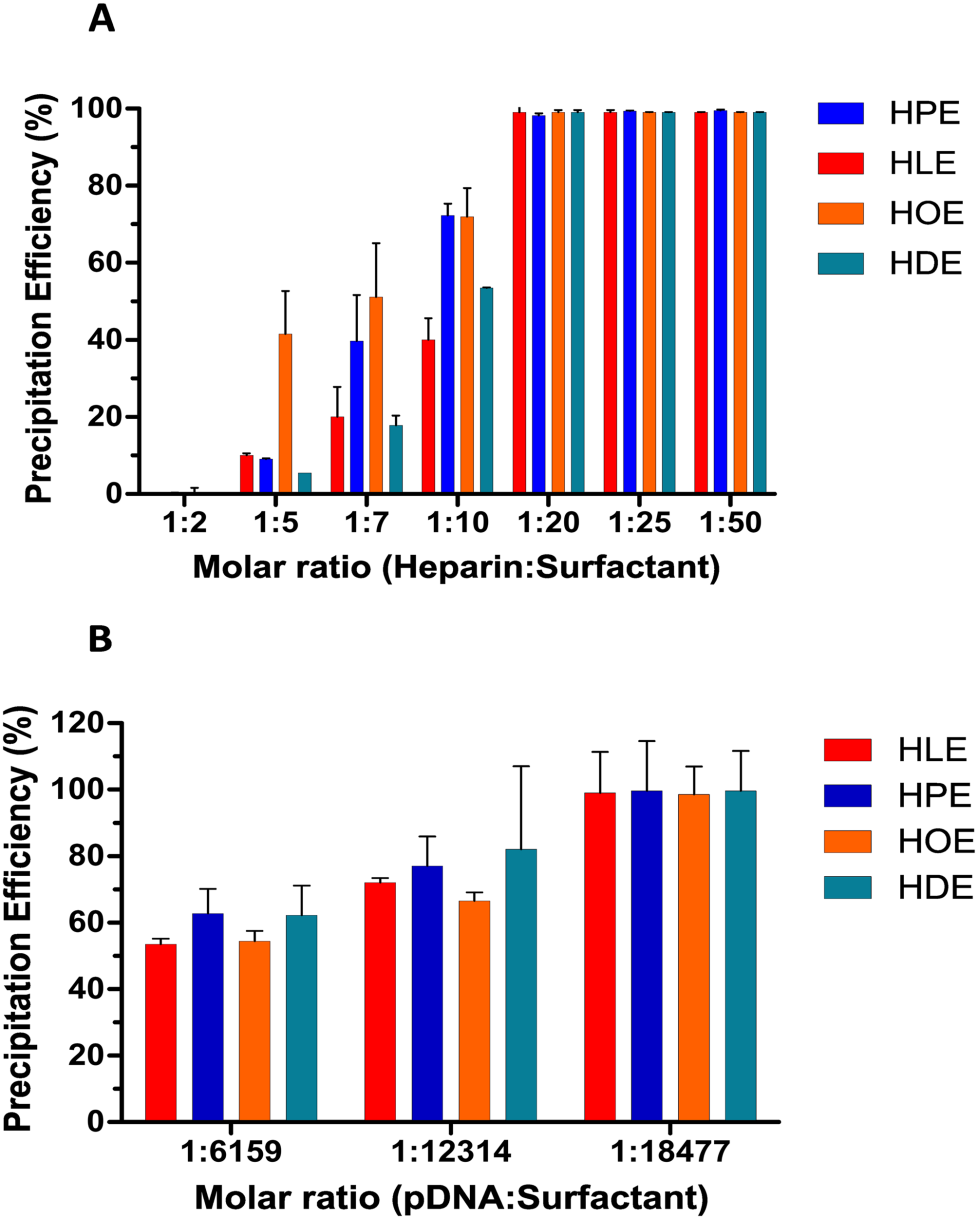
**A** Percentage precipitation of heparin with HLE, HPE, HOE, and HDE at indicated surfactant to heparin molar ratios. **B** Percentage precipitation of pDNA with HLE, HPE, HOE, and HDE at indicated surfactant to nucleic acid molar ratios. Data are shown as mean ± SD (n = 3)

The potential of His-surfactants to form hydrophobic ion pairs with macromolecular drugs and nucleic acids was evaluated using heparin and pDNA as model drugs. The efficiency of His-surfactants as hydrophobic ion pairing agents was evaluated using molar ratios ranging from 1:2 to 1:50 (heparin:surfactant) and from 1:6159 to 1:18477 (DNA:surfactant). The heparin-surfactant complex formation increased with increasing the molar ratio from 1:2 to 1:20 (Fig. 6A) for each compound. All formed HIPs were obtained as white solids except for heparin-HOE which appeared as a yellowish oily phase. Heparin-HOE complexation yields higher precipitation efficiencies in the range of 1:5 − 1:10, likely due to its higher hydrophobicity. Overall, each His-surfactant yielded 100% precipitation efficiency at the molar ratio of 1:20, resulting in higher efficiency than the corresponding arginine-based surfactants [28]. Theoretically, the maximum precipitation efficiency can be reached when every negative charge is paired with a positive charge. Therefore, a molar ratio of 1:6159 (pDNA:surfactant) was applied, referring to the stoichiometric ratio of charged moieties. On the other hand, the maximum precipitation efficiency could be obtained only at a molar ratio of 1:18477 (pDNA:surfactant) (Fig. 6B). This observation might be explained by additional effects influencing precipitation efficiency such as an increase in ionic strength and/or non-ionic interactions between the drug and the counterion or counterion-counterion, as already reported by other authors [27, 50–52]. The complexation with His-surfactants allows complete heparin and pDNA complexation and precipitation without any complex re-solubilization due to micelle formation. The obtained results confirm the hypothesis that these compounds can effectively be employed as counterions for hydrophobic ion pairing with hydrophilic macromolecular drugs like heparin and nucleic acids.

### Characterization of HIPs

**Fig. 7:**
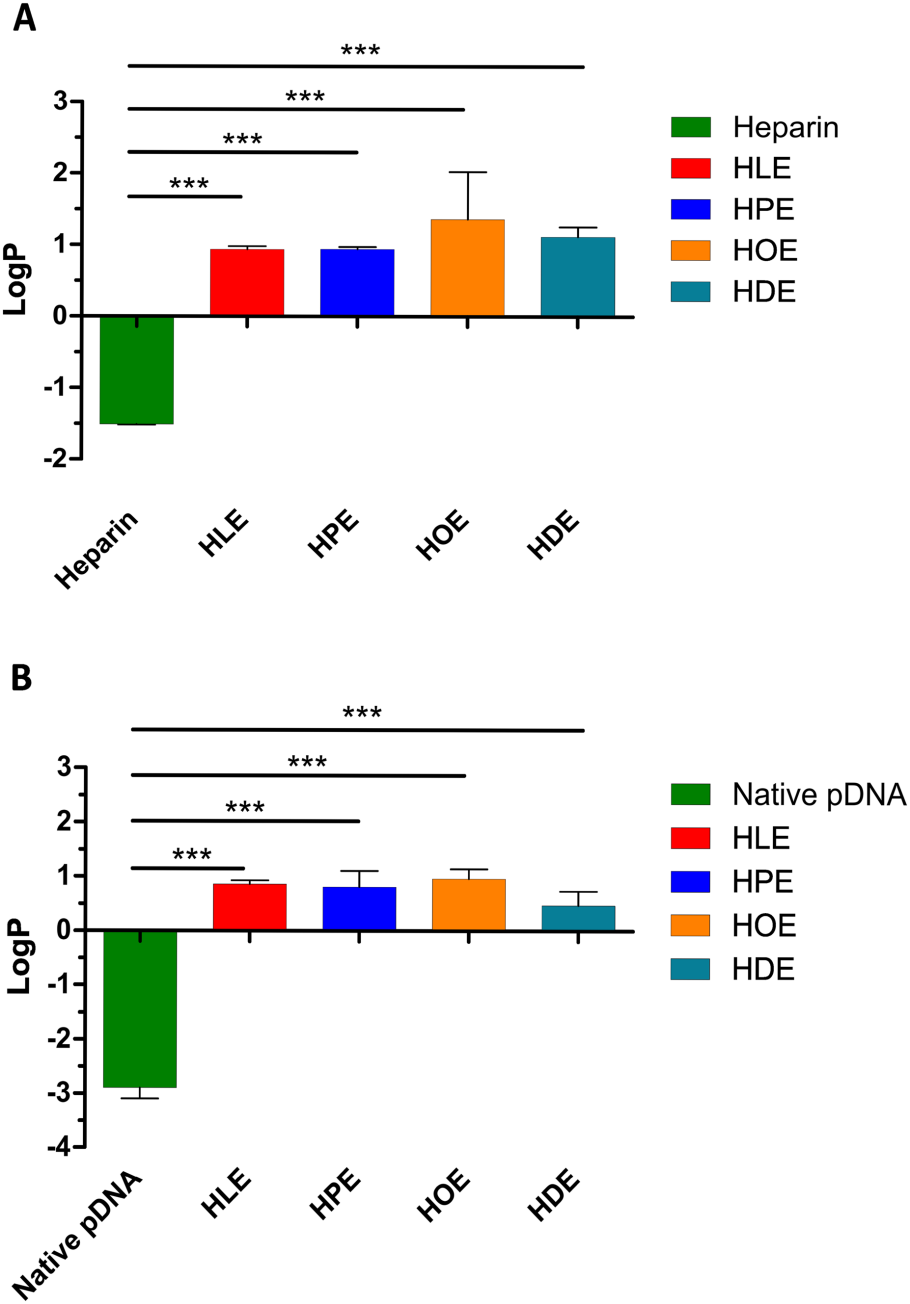
**A** LogP values (octanol/water partition coefficients) of free heparin and HIPs with His-surfactants. **B** LogP values (octanol/water partition coefficients) of free pDNA and HIPs with His-surfactants. Data are shown as mean ± SD (n = 3)

Heparin and pDNA HIPs were further characterized regarding their increase in lipophilicity via evaluation of the partition coefficient water/octanol. The logP of heparin and pDNA was effectively increased by HIP formation with His-surfactants, leading to the formation of a white precipitate in aqueous media. The lipophilicity of heparin and pDNA was increased by more than 300-fold and 3000-fold, respectively (Fig. 7A, B), enabling their incorporation into lipid-based formulations. In comparison to previous arginine-based surfactants investigated as counterions for heparin HIP [28] (i.e., arginine nonyl ester and arginine hexadecanoyl ester), the complexes obtained with His-surfactants investigated within this study showed higher lipophilicity. Moreover, in the case of pDNA, HIP allowed to reverse the hydrophilic character of the nucleic acid to lipophilic. Overall, considering the obtained data, increased complex solubilization in lipophilic vehicles employed in lipid-based formulations is expected.

**Fig. 8:**
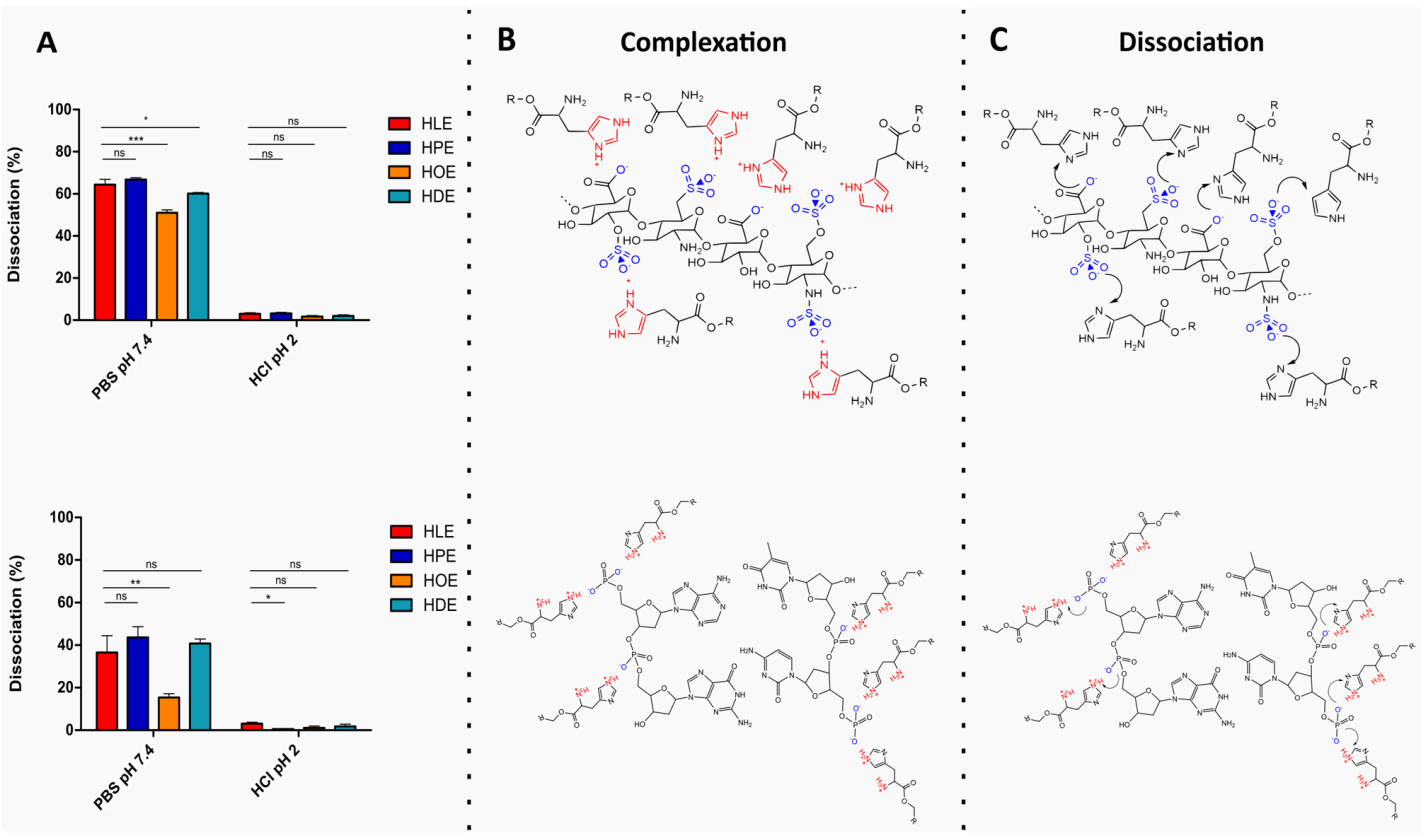
(**A**; upper) Dissociation study of heparin HIPs with HLE (red bars), HPE (blue bars), HOE (orange bars), and DHE (turquoise bars) at indicated pH at 37 °C, 300 rpm for 4 h. (**A**; lower) Dissociation study of pDNA HIPs with HLE (red bars), HPE (blue bars), HOE (yellow bars) and DHE (turquoise bars) at indicated pH at 37 °C, 300 rpm for 4 h. (**B**; upper) HIP structure during complexation of heparin with each surfactant. (**B**; lower) HIP structure during complexation of pDNA with each surfactant. (**C**; upper) HIP structure during dissociation of heparin with each surfactant. (**C**; lower) HIP structure during complexation of pDNA with each surfactant. Data are shown as mean ± SD (n = 3)

The stability of the newly formed HIPs was evaluated by determining the dissociation behavior under physiological conditions at pH 7.4 and 2.0 (Fig. 8A). At physiological pH, > 50% heparin HIP dissociated within 4 h. On the contrary, the complexes proved to be highly stable under acidic conditions. The same behavior was observed in the case of pDNA HIP. This behavior can be attributed to the pH-dependent charge of His-surfactants. At pH 2.0 the imidazolium function is protonated, and His-surfactants retain their ability to complex heparin and pDNA, but above the pK_a1_ (pH 7.4), the imidazole does not bear a positive charge anymore, resulting in an increased dissociation of the HIPs. The observed behavior of the investigated HIPs confirms their suitability for drug delivery purposes, as HIPs, once delivered to the site of action, should dissociate in order to allow the drug to exert its function. According to the obtained data, a dissociation under acidic gastrointestinal conditions is not favored. However, PBS, mimicking physiological conditions, can trigger dissociation, thereby enabling the release of the hydrophilic therapeutic agent.

## Conclusion

Within this study, the amino-acid histidine (His) was successfully coupled with naturally occurring fatty alcohols (lauryl, palmitoyl, oleyl) and 2-hexyl-1-decanol via ester bond formation. The obtained surfactants demonstrated the ability to be cleaved by an endogenous enzyme represented by trypsin within 24 h and low cytotoxicity on Caco-2 cell line, representing an eco- and patient-friendly alternative to the commonly employed cationic surfactants. The investigated His-surfactants displayed favorable characteristics regarding hydrophobic ion pair formation with the hydrophilic macromolecular drug heparin and plasmid DNA. Complexation led to complete drug precipitation in aqueous media, and the resulting complexes exhibited increased lipophilicity. According to the obtained results, the formed His-complexes are promising candidates for incorporation in lipid-based drug delivery systems for oral delivery of heparin and nucleic acids. SEDDS might be employed for this purpose, due to their favorable properties in terms of protection against the harsh gastrointestinal environment, as well as mucus permeating properties. Considering the encouraging outcome of this study, biodegradable His-surfactants might represent a valuable alternative to the established surfactants not only in the pharmaceutical field but also for food and cosmetic industry.

## Data Availability

The authors confirm that the data supporting the findings of this study are available within the article and its supplementary information files. The datasets generated during or analyzed during the current study are available from the corresponding author on reasonable request.

## Supporting Information

Below is the link to the electronic supplementary material.

Supplementary Material 1
