# Supplementary material for: Histidine-based ionizable cationic surfactants: novel biodegradable agents for hydrophilic macromolecular drug delivery
Source: Drug Deliv Transl Res. 2024 Jan 30;14(9):2370–85. doi: 10.1007/s13346-023-01511-8 (PMC11291603; doi:10.1007/s13346-023-01511-8)
Supplement: Supplementary file 1 — Supplementary Material 1 [file 13346_2023_1511_MOESM1_ESM.docx]

Histidine-based ionizable cationic surfactants: novel biodegradable agents for hydrophilic macromolecular drug delivery

Ilaria Polidori^1^, Dennis To^1^, Gergely Kali^1^, Andreas Bernkop-Schnürch^1^*

Department of Pharmaceutical Technology, University of Innsbruck, Institute of Pharmacy, Center for Chemistry and Biomedicine, 6020 Innsbruck, Austria

*Corresponding author

^1^Department of Pharmaceutical Technology, University of Innsbruck, Institute of Pharmacy, Center for Chemistry and Biomedicine, 6020 Innsbruck, Austria

Tel.: +43 512 507 58 600

Fax: +43 512 507-58699

Email: [Andreas.Bernkop@uibk.ac.at](mailto:Andreas.Bernkop@uibk.ac.at)

# Supplementary materials


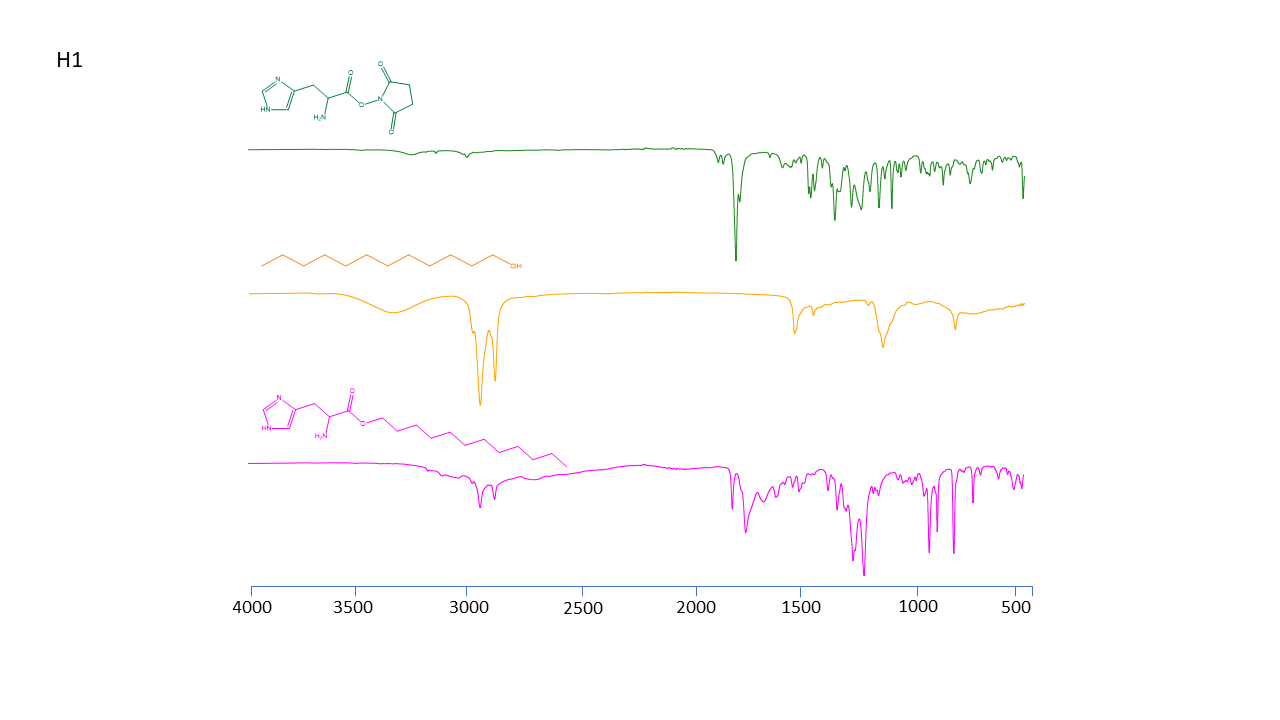


**Fig. S1** FT-IR spectra HLE.


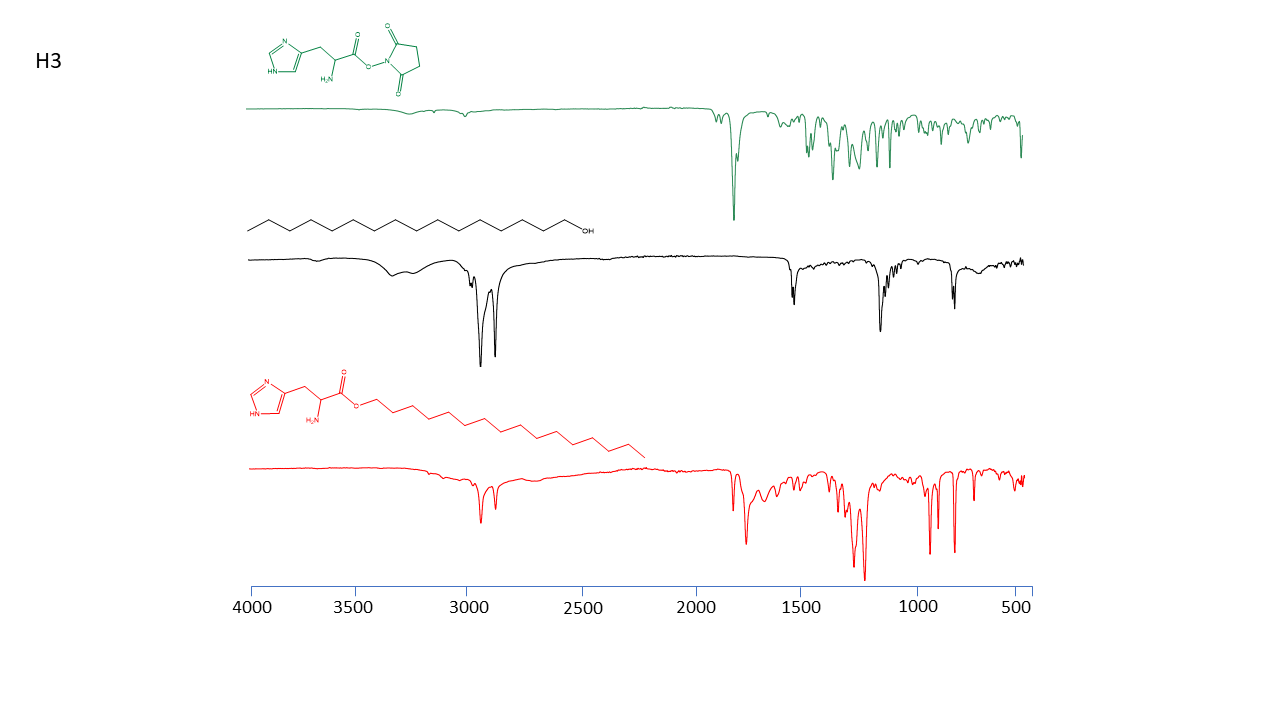


**Fig. S2** FT-IR spectra HPE.


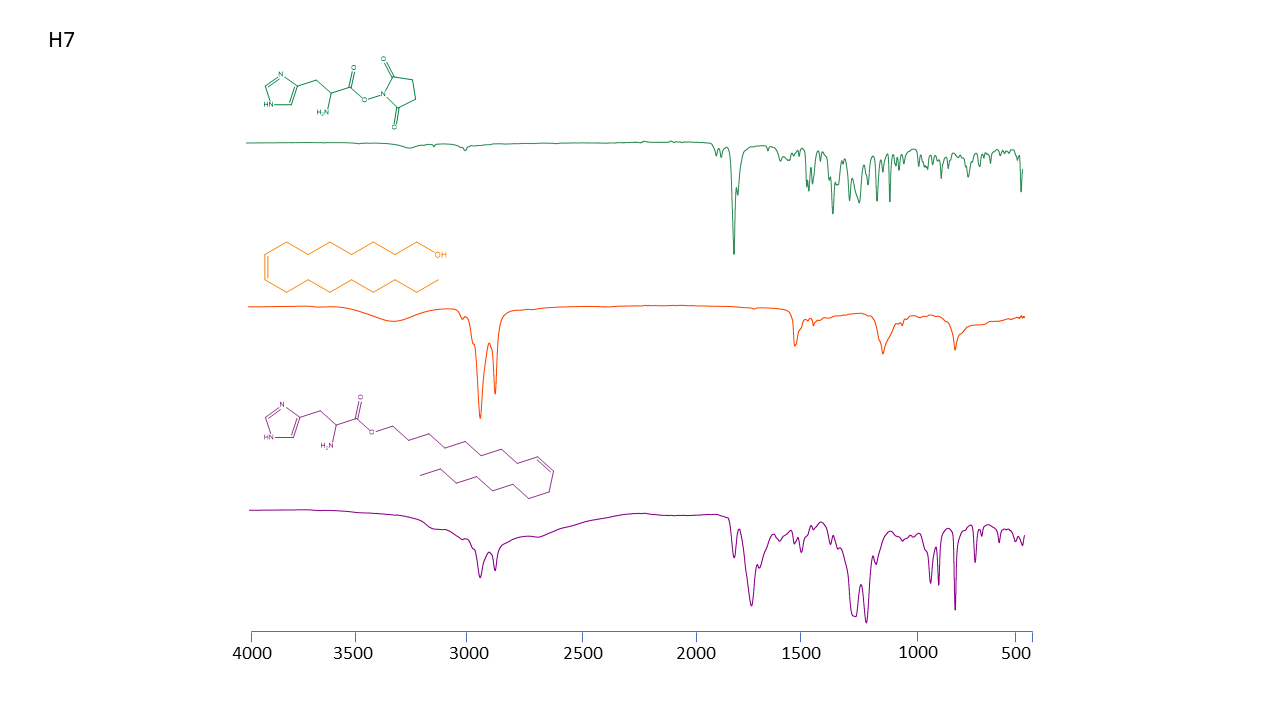


**Fig. S3** FT-IR spectra HOE.


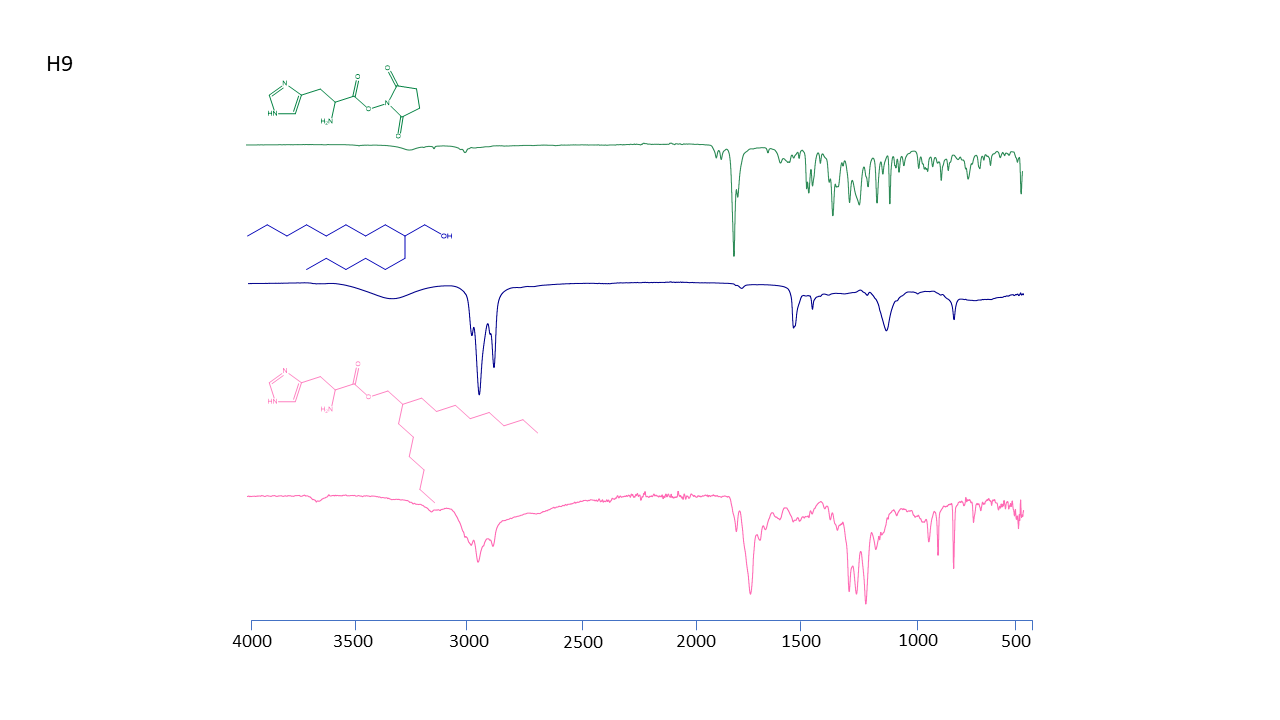


**Fig. S4** FT-IR spectra HDE.


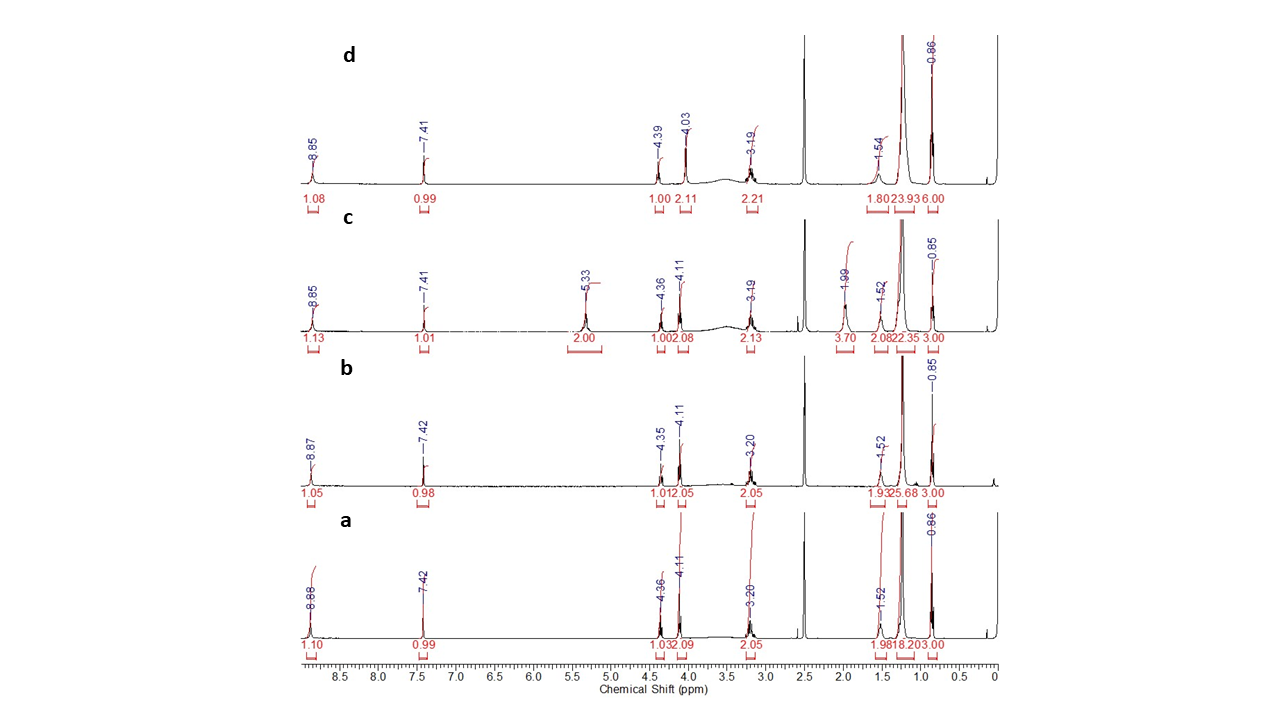


**Fig. S5** 400 MHz ^1^H NMR spectra of (a) HLE, (b) HPE, (c) HOE, and (d) HDE in DMSO-*d_6_*.


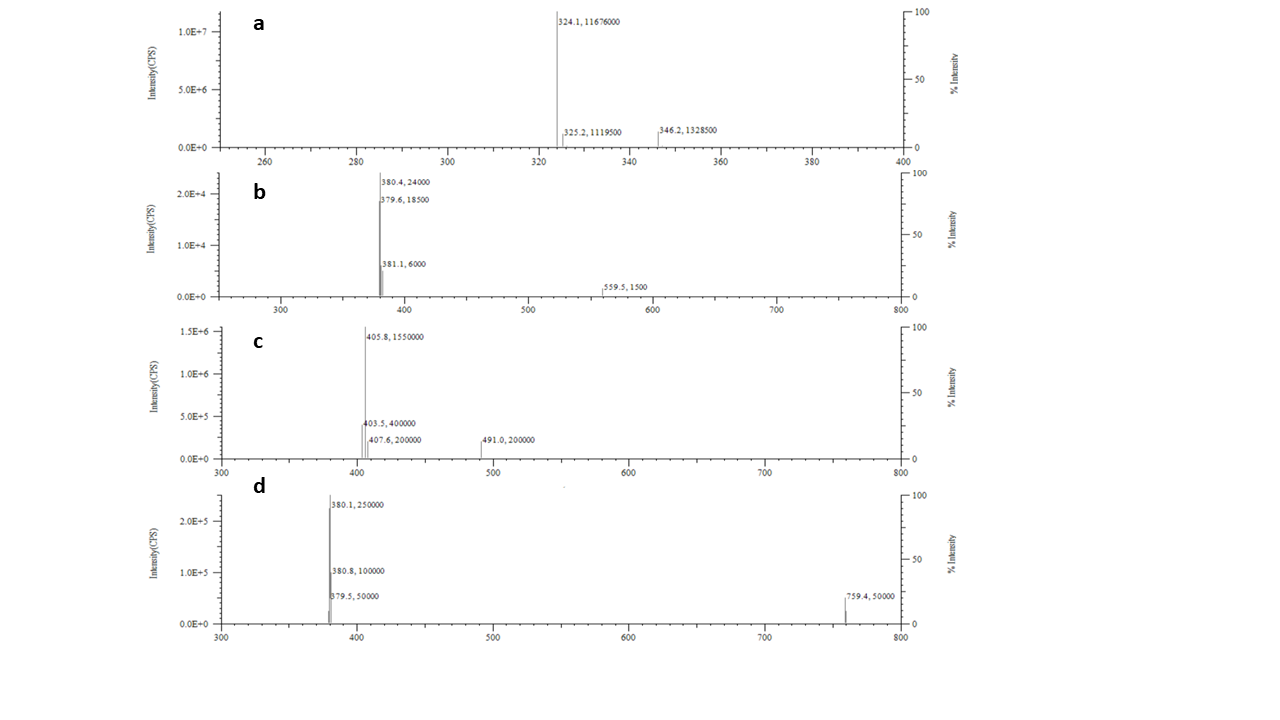


**Fig. S6** Mass spectrum of (a) HLE, (b) HPE, (c) HOE and (d) HDE.


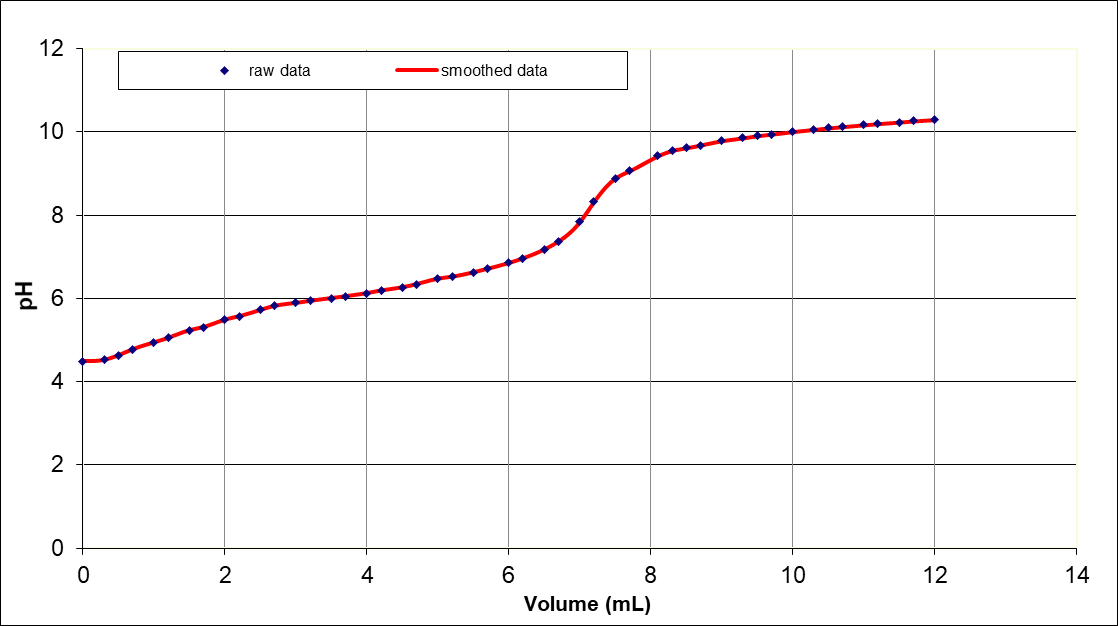


**Fig. S7** pK_a_ HLE.


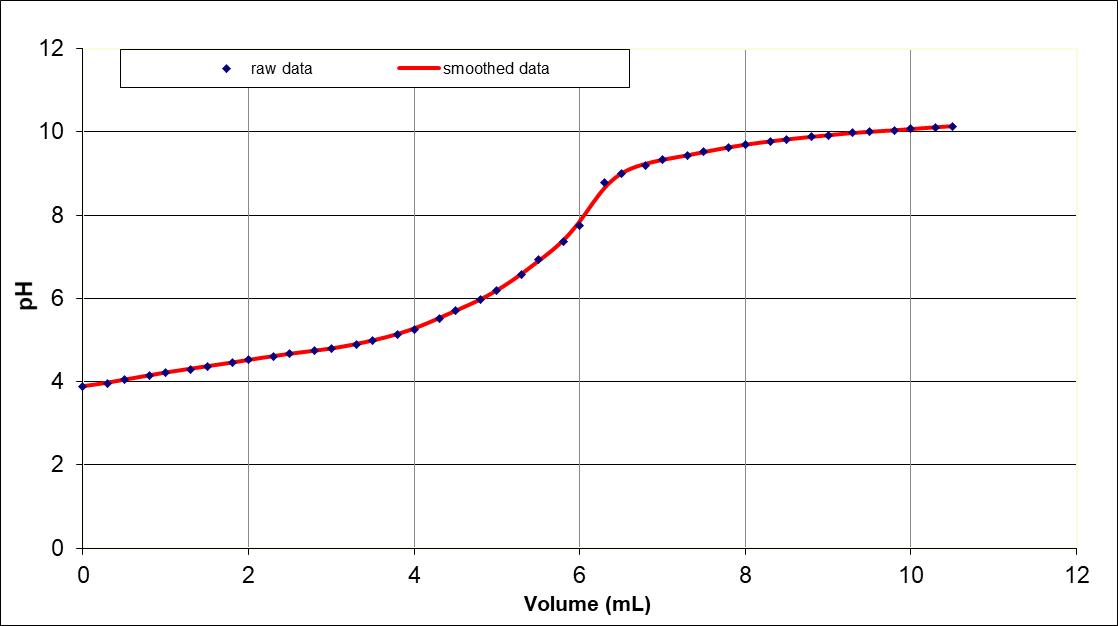


**Fig. S8** pK_a_ HPE.


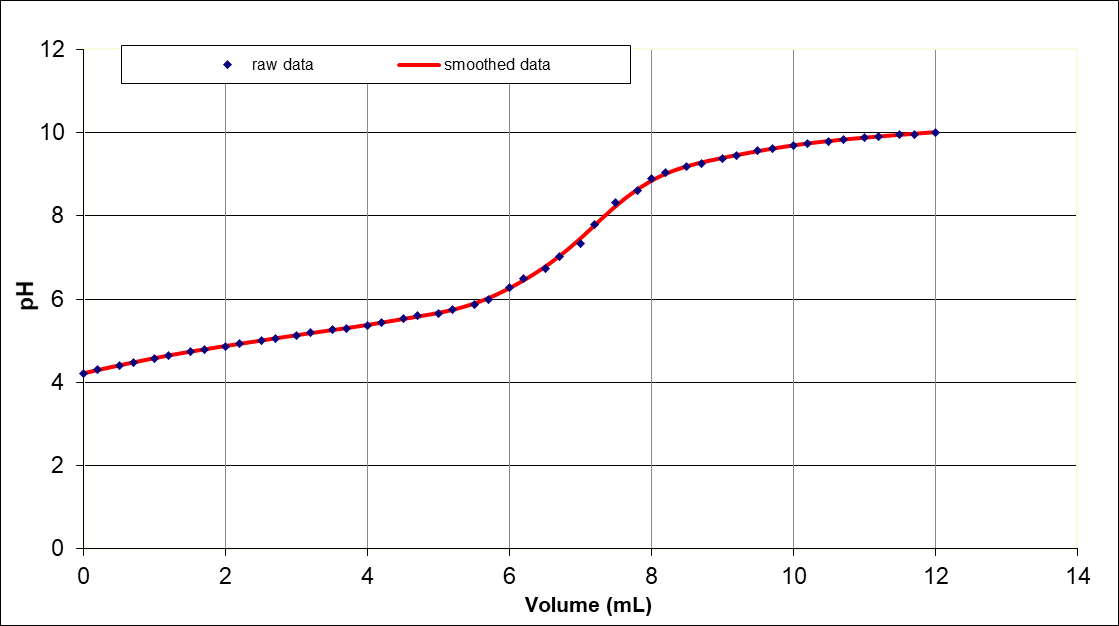


**Fig. S9** pK_a_ HOE.


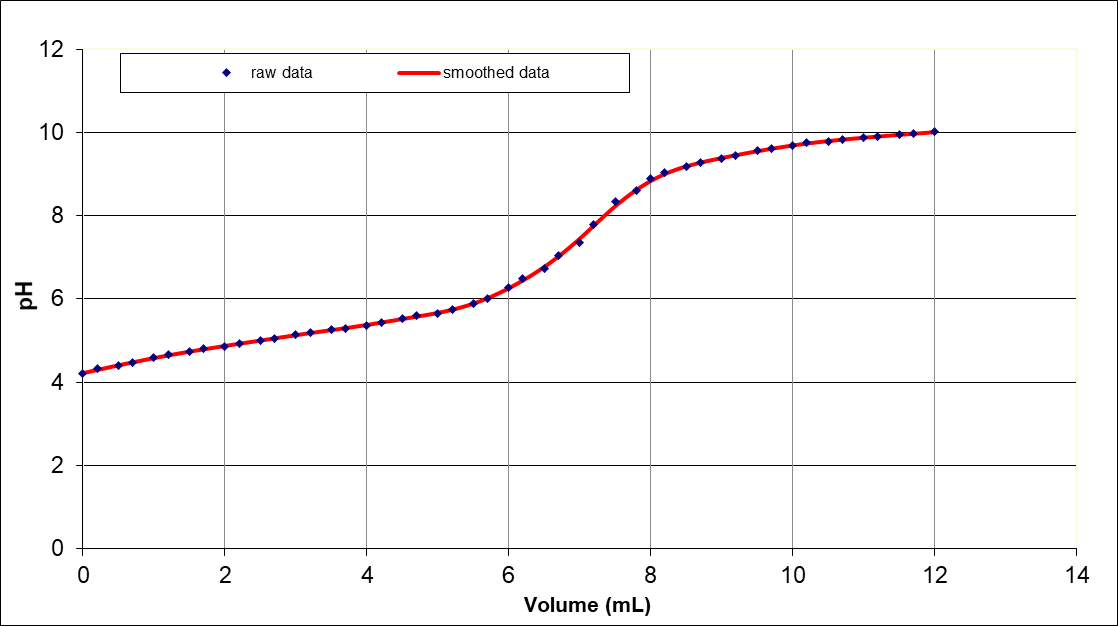


**Fig. S10** pK_a_ HDE.
